# Association of Arrhythmias in Cardiac Amyloidosis and Cardiac Sarcoidosis

**DOI:** 10.7759/cureus.9842

**Published:** 2020-08-18

**Authors:** Ibtisam Ashraf, Mercedes Maria Peck, Ruchira Maram, Alaa Mohamed, Diego Ochoa Crespo, Gurleen Kaur, Bilal Haider Malik

**Affiliations:** 1 Internal Medicine, Shalamar Institute of Health Sciences, Lahore, PAK; 2 Internal Medicine, California Institute of Behavioral Neurosciences and Psychology, Fairfield, USA; 3 Internal Medicine, California Institute of Behavorial Neurosciences and Psychology, Fairfield, USA; 4 Internal Medicine, Arogyasri Healthcare Trust, Hyderabad, IND; 5 Internal Medicine, Memorial Hermann Medical Center, Houston, USA; 6 Internal Medicine, Clinica San Martin, Azogues, ECU

**Keywords:** cardiac amyloidosis, cardiac sarcoidosis, arrhythmia, catheter ablation, implantable cardioverter-defibrillator, atrial arrhythmia

## Abstract

Cardiac involvement in amyloidosis and sarcoidosis is poorly understood, and is associated with high morbidity and mortality. Atrial and ventricular arrhythmias, along with conduction defects, are frequent in cardiac amyloidosis and sarcoidosis. Atrial dysfunction in cardiac amyloidosis may result in atrial fibrillation and increases the risk of stroke, making anticoagulation significant and challenging. Ventricular arrhythmia and conduction defects are more common in AL amyloidosis and cardiac sarcoidosis. Premature ventricular contractions (PVCs) from Purkinje fibers trigger ventricular arrhythmias in cardiac amyloidosis, while the inflammation and scarring leading to the reentrant process is the cause in cardiac sarcoidosis. The typical treatment modalities include Class II and III antiarrhythmic drugs and ablation techniques, while corticosteroids and immunosuppressants are indicated in cardiac sarcoidosis to reduce the burden of the disease and arrhythmias. Sudden cardiac death can be a manifestation of both disorders that can be prevented by the Implantable cardioverter-defibrillator (ICD), although the predictive risk factors for primary prevention remain uncertain. In this review, we addressed the current understanding of the pathways involved in inducing arrhythmias in cardiac amyloidosis and sarcoidosis-also, the complications including sudden death and stroke associated with arrhythmia in both diseases. We have discussed other preventive steps needed to minimize arrhythmias to provide symptomatic relief and palliation to patients.

## Introduction and background

Cardiac amyloidosis (CA) and sarcoidosis are typically known as infiltrative cardiomyopathies. The incidence of cardiac amyloidosis is 18 per 100,000 person-years, while cardiac sarcoidosis occurs in 5% of the patients with systemic sarcoidosis (10-20 per 100,000 individuals) [1,2]. Amyloidosis is characterized by the deposition of insoluble amyloid fibrils in extracellular tissues involving multiple body systems, including cardiac tissue. Several forms of precursor protein significantly affect the heart: light-chain (LC) immunoglobulin, mutant hereditary transthyretin (TTR), wild-type TTR, mutant apolipoprotein AI, amyloid atrial natriuretic peptide localized to the atrium, fibrinogen alpha type and serum amyloid A protein [3]. Sarcoidosis is a granulomatous multisystem disease in which CD4+ T-lymphocytes aggregate to induce a Th-1 type immune response [4]. Both diseases may involve cardiac tissue either as a part of the systemic condition or in isolation. Heart failure, conduction abnormalities, and arrhythmias are the most common signs of cardiac involvement [1,4]. Arrhythmias in cardiac amyloidosis vary by amyloidosis type, as conduction defects and supraventricular arrhythmias are more prevalent in transthyretin amyloidosis [5]. Atrioventricular (AV) block is the most common type of arrhythmia in cardiac sarcoidosis, followed by ventricular tachycardia and supraventricular arrhythmia [6].

Cardiac involvement in systemic sarcoidosis and amyloidosis typically results in poor prognosis. They account for around 10-25% of all sarcoidosis deaths in the US and are generally due to malignant arrhythmias and complete heart block [7]. The precise cause of arrhythmias in CA is less clearly known and is likely to be multifactorial [5]. There is little understanding of the exact mechanism of sudden cardiac arrest and the identification of CA patients that may be eligible for implantable cardioverter-defibrillator (ICD) [8]. Whether implantable cardioverter-defibrillator (ICD) use in such patients prevents sudden cardiac death (SCD) is uncertain. Furthermore, atrial dysfunction in amyloidosis raises the likelihood of intracardiac thrombus formation that can lead to stroke, a probability unknown [9]. Ventricular arrhythmias arising in cardiac sarcoidosis can be fatal and its mechanism and prevalence are not well established. Glucocorticoids are the mainstays of treatment in cardiac sarcoidosis, but data from randomized controlled studies are scarce. Similarly, the precise role of corticosteroids in managing arrhythmias including atrioventricular blocks (AVBs) needs to be established.

These disorders have diverse clinical manifestations and severe outcomes that render it challenging to diagnose and manage. Also, there is little understanding of electrophysiological abnormalities in infiltrative cardiomyopathies. This review aims to address the cause, relationship, and complications of arrhythmias, based on the most current data, in cardiac amyloidosis and sarcoidosis.

## Review

Pathogenesis of arrhythmias in cardiac amyloidosis and cardiac sarcoidosis

Cardiac amyloidosis (CA) results from the infiltration of the extracellular spaces by amyloid, isolating, and misshaping the structure of myocardial cells. This deposition increases ventricular wall thickness resulting in restrictive cardiomyopathy [10]. Electrocardiogram (ECG) often shows low voltage QRS complex and pseudo infarct patterns on precordial leads. Several different mechanisms aid myocardial dysfunction. In addition to infiltration, light chains in AL amyloidosis may also induce direct toxicity by increasing the intracellular reactive oxygen species [11]. Evidence of amyloid accumulation is found in perivascular regions and media of intramyocardial coronary vessels that may induce coronary microvascular dysfunction leading to ischemia and hence to myocardial injury [12]. Studies in Sweden, involving cases of familial amyloidosis with polyneuropathy, have specifically demonstrated amyloid infiltration of the sinus node and atrioventricular conduction system [13,14]. Consequently, this inflammatory cell injury, structural destruction, and separation of myocytes by amyloid fibrils explain the electrophysiological abnormalities.

Cardiac sarcoidosis (CS) is a granulomatous disease that results in conduction abnormalities and ventricular arrhythmias. The precise cause of sarcoidosis is unclear, however, it seems to affect the basal septum, atrioventricular node (AV node), atrioventricular (His) bundle, the focal regions of the ventricular free walls, and the papillary muscles of the heart [15]. Isolated cardiac sarcoidosis accounts for two-thirds of all cardiac sarcoidosis cases, requiring the diagnosis of CS and the absence of sarcoid involvement of any other organ. Electrocardiograms can exhibit atypical infarction patterns and atrioventricular (AV) blocks, as shown in Table 1. This cardiac disease typically progresses from localized inflammation to scarring, resulting in atrial and ventricular arrhythmias. Similarly, inflammatory processes tend to include basal interventricular septum and result in atrioventricular blocks (AVB) and bundle branch blocks. In a retrospective study in Finland, atrioventricular blocks were found to be the most frequent presenting symptom in cardiac sarcoidosis, arising in about 44% of the cases. In contrast, ventricular arrhythmias were the second most common presenting symptom, occurring in 33% of the cases [6].

**Table 1:** Comparison between Cardiac Amyloidosis and Cardiac Sarcoidosis AV = atrioventricular; SCD = sudden cardiac death; CHB = complete heart block; LGE = late gadolinium enhancement

	Age at Presentation	Clinical Presentation	Echocardiography	ECG	CMR LGE	Biopsy
Cardiac Amyloidosis	6^th^ or 7^th^ decade, Male > Female, ATTR variant common in African American	Heart Failure, Nephrotic syndrome, Peripheral neuropathy	Increased LV and RV wall Thickness, Normal or small LV cavity size, biatrial enlargement, granular appearance of myocardium	Low-voltage QRS complex, Pseudo-infarct pattern in precordial leads	Global transmural or subendocardial LGE	Replacement of normal cardiac tissue by amyloid
Cardiac Sarcoidosis	3^rd^ or 4^th^ decade, Female > Male, More common in African Americans	Heart Failure, SCD, CHB	Segmental wall motion abnormalities and Variable Septal Thickness	AV Block/Infrahisian block, Atypical Infarction Pattern	Patchy basal and LV free wall	Noncaseating multinucleated giant cell granulomas

Atrial arrhythmias

Atrial fibrillation (AF) and atrial tachycardia (AT) are the most commonly occurring arrhythmias in cardiac amyloidosis. The combination of intramyocardial amyloid accumulation, leading to restricted ventricular relaxation and elevated filling pressures, which eventually causes left atrial enlargement, and the intrinsic left atrial dysfunction due to direct amyloid deposition, are the causes of atrial dysfunction in CA [16]. Atrial fibrillation is more frequent in wild-type transthyretin amyloidosis (ATTRwt) [5]. Barbhaiya et al. performed an atrial arrhythmia substrate analysis, which showed supraventricular tachycardia in all 18 patients with CA [17]. Another study showed that both interventricular septum (IVS) thickness and atrial dimension were associated with an increased rate of supraventricular arrhythmia [18]. Amyloid accumulation in atria isolates atrial myocyte bundles, which is why AF in CA appears to have longer cycle lengths because the mechanism induces a pronounced delay in intra-atrial conduction [5].

The precise prevalence of atrial arrhythmias in cardiac sarcoidosis is unclear. Atrial arrhythmias, like AF/AT, are less frequent than ventricular arrhythmias in CS. Viles-Gonzalez et al. documented supraventricular arrhythmia in 32% of CS cases (atrial fibrillation 18%, atrial tachycardias 7%, atrial flutter 5%, and other types of supraventricular tachycardias 2%) [19]. In contrast, Cain et al. evaluated 192 patients with extracardiac sarcoidosis (confirmed with a biopsy and CMR imaging) and found that atrial arrhythmias (36%) were more common than ventricular arrhythmias (27%) [20]. CS induces inflammation and scarring of the atrial tissue and atrial enlargement due to ventricular dysfunction that leads to atrial arrhythmias. Autopsy of patients with cardiac sarcoidosis showed sarcoidosis lesions in the left ventricular free wall (96%), the ventricular septum (73%), the right ventricular wall (46%), the right atrium (11%), and the left atrium (7%) [21]. Multivariate analysis showed that left atrial enlargement was the only independent factor consistent with atrial arrhythmias in CS patients [19].

Atrial thrombus formation in CA

Cardiac amyloidosis raises the risk of the development of intracardiac thrombus, which can result in cardioembolic stroke. A study showed that 33% of the 116 autopsy cases had intracardiac thrombi and that the potential cause may be the combination of hypercoagulability, endomyocardial disruption, and endothelial dysfunction due to amyloid deposition [22]. Amyloid accumulation causes atrial mechanical dysfunction that can lead to thrombus formation even in the absence of atrial arrhythmia [9]. In another study, intracardiac thrombus was shown more frequently in AL amyloid than in other forms (35% vs. 18%) [23]. Stroke in CA is not an uncommon complication that can occur even in patients on anticoagulation therapy. Recently, 8.9% of the 89 patients with CA had an ischemic stroke [24]. Thus, it is crucial to estimate the correct risk of stroke in CA patients with or without atrial fibrillation.

Ventricular arrhythmias

Ventricular arrhythmias are more frequently observed in light-chain (LC) immunoglobulin (AL) amyloidosis, mainly due to its steep downward path following the onset of heart failure [5]. Dubery et al. examined the 24-hour Holter recordings of 195 patients with AL amyloidosis and found non-sustained ventricular tachycardia (VT) in 27% of patients [25]. In another study with implanted loop monitors, bradycardia was reported in 20 patients with AL CA death, and there was only one episode of non-sustained ventricular tachycardia, preceded by severe bradycardia [26]. Ventricular fibrillation may be caused by premature ventricular contractions (PVC) originating from the Purkinje system. It can occur in post-MI patients as well as in chronic ischemic cardiomyopathy. A study reported that two CA patients had repetitive VF episodes with each episode preceded by monomorphic PVC [27]. Nevertheless, the precise cause of VF arising in CA is not well understood.

Ventricular tachycardia (VT) is the second most frequent form of arrhythmia in cardiac sarcoidosis. Ventricular arrhythmias in CS are due to reentrant and focal (triggered activity or abnormal automaticity) mechanisms. The scarred areas of ventricular endocardium and epicardium are capable of sustaining a significant number of reentrant circuits and are niduses for re-entry [28]. Naruse et al. reported a sequence of 37 patients with CS in which a total of 57 VTs were induced; 14 VTs were non-sustained, six were polymorphic, six were associated with Purkinje system, and 31 were associated with scar areas [29]. Sustained monomorphic VT in CS is a significant indicator of mortality. It can lead to hemodynamic collapse, which means that these patients need an immediate conversion to sinus rhythm. Also, the risk stratification of ventricular arrhythmias in these patients is necessary.

Conduction defects

Cardiac involvement in amyloidosis may cause conduction anomalies that are evident to ECG as the narrow QRS complex, bundle branch blocks, fascicular blocks, and atrioventricular blocks (AVB). A recent study has shown that ventricular conduction and repolarization defects are higher in AL relative to ATTR amyloidosis [30]. Conduction abnormalities such as atrioventricular conduction delay are more common than sinus node disease, despite the frequent atrial involvement in CA. Intracardiac electrophysiology (EP) examination of sinus node function in 25 patients with AL amyloidosis showed normal sinus node function in 88% of the patients tested [31]. On the other hand, a previous study showed that a common morphological abnormality of the conduction system was severe sinoatrial fibrosis [32]. However, patients with cardiac amyloidosis have frequent dysfunction in the His-Purkinje system. It is demonstrated with HV prolongation, which is the conduction time measured from the onset of His potential to the beginning of intracardiac ventricular activation. Reisinger et al. showed that the mean HV interval in 25 patients was 79 +/-18 ms and 23 patients (92%) had an abnormally prolonged interval (>55 ms) while 12 patients showed a pronounced HV prolongation (>80 ms), six of which had an interval >100 ms [31]. This suggests a significant infiltration of myocardial tissue, including the conduction system, by amyloid fibrils. Such patients also have a high frequency of sudden death, the HV interval being an independent indicator.

**Figure 1:**
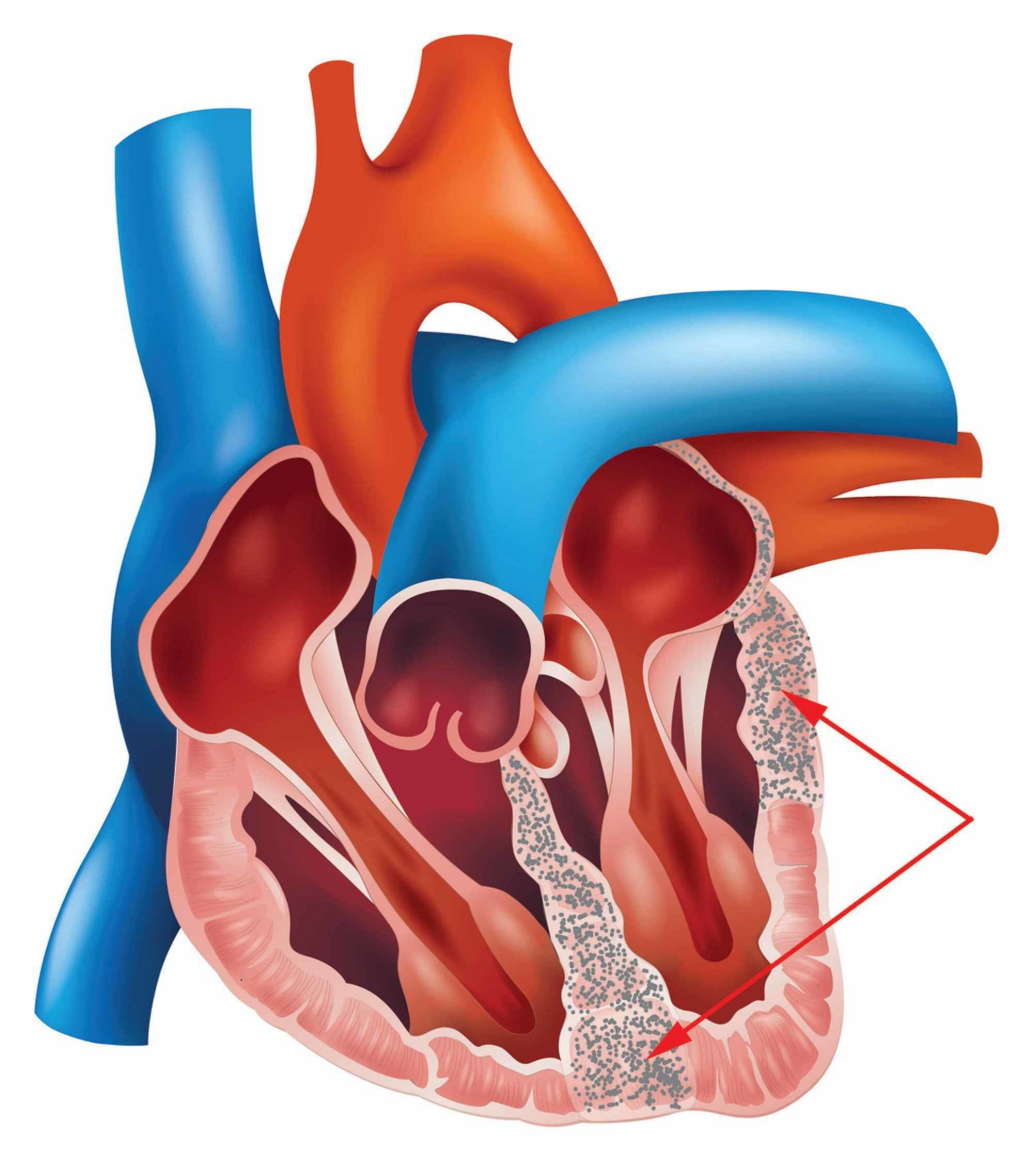
Showing Septum and LV fibrosis in sarcoidosis clinically manifested as Heart Block, Heart failure, and Ventricular Tachycardia due to reentrant circuits around the scar.

Conduction disorders, including atrioventricular blocks (AVBs), are the most common manifestations of cardiac sarcoidosis, responsible for around half of the cases. The primary cause of this is widespread inflammation and fibrosis in the conduction system, as shown in Figure 1. The right bundle branch block is frequent and can be a symptom of newly developed CS in documented extracardiac sarcoidosis patients. A study showed that 34% of patients 60 years of age with AVB had CS as an underlying condition [33]. Another study found that sudden death was the initial manifestation of sarcoidosis in 10/60 patients (17%) who died unexpectedly, while complete heart block was the most prevalent conduction disorder in 25 patients, and a complete bundle branch block occurred in 21 patients [21]. Therefore, all patients <60 years of age who present with high-degree AV block should be screened for CS. 

Management of arrhythmias

The management of arrhythmias in cardiac amyloidosis and sarcoidosis is a complicated process. The prognosis of both conditions is generally poor, however, antiarrhythmic and immunosuppressive therapy and cardiac ablation techniques are increasingly being used to control the underlying arrhythmias. Implantable cardioverter-defibrillator (ICD) can be used to prevent sudden death due to fatal arrhythmias in these cases.

Antiarrhythmic and Immunosuppressive Therapy

The evidence on antiarrhythmic medications is limited. Class III antiarrhythmic medications are used to regulate AF/AT in cardiac amyloidosis but are ineffective in cardiac sarcoidosis. Corticosteroid treatment has proven successful in reducing the burden of AF in CS [2]. Anticoagulation is strongly recommended for cardiac amyloidosis patients with atrial fibrillation due to an increased risk of stroke.

Similarly, class III agents are also used to treat ventricular arrhythmias in cardiac sarcoidosis. However, amiodarone can induce pulmonary toxicity in these patients and may be deferred in advanced pulmonary sarcoidosis [2]. Corticosteroid therapy has been effective in treating ventricular arrhythmias and improving AV conduction in patients with CS. A study reported a 47% improvement in AV conduction in patients treated with corticosteroids, with no improvement in the untreated group [34]. Another study showed that the patients who received early treatment with corticosteroids resulted in no ventricular arrhythmia recurrences in 8/11 patients (72%), and complete recovery of AV conduction in 2/3 patients (67%) [35]. In addition, methotrexate and azathioprine can be used as an alternative to corticosteroids in these cases.

Ablation

AV node ablation is usually done in CA and CS patients with atrial arrhythmia (AF) and with persistent symptoms despite the optimal medical therapy. It can improve the rate and restore sinus rhythm, providing symptomatic relief in patients with atrial fibrillation. However, there is an increased rate of recurrence in CA patients with AF. A study showed a one-year recurrence rate of 83% in CA patients compared to 25% in non-CA patients [5]. Whereas CS patients with AF had a recurrence rate of 22% (2/9 patients) following ablation [36].

Ventricular arrhythmias in CA are caused by PVC arising from the distal Purkinje system. Thus, catheter ablation of the triggering PVC from the Purkinje system can be used as a therapy in these patients. Mlcochova et al. identified two patients with repetitive VF related to cardiac amyloidosis [27]. PVC followed each episode of ventricular arrhythmia, which was not associated with the scar tissue but was drug-resistant. The recurrence of VF was successfully eliminated by catheter ablation.

On the other hand, ventricular arrhythmia in CS is due to a reentrant mechanism and is associated with scar tissue. A systematic review was done involving 83 patients and five studies [37]. The mean age of patients was 50 ± 8 years, and the mean ejection fraction was 39.1 ± 3.1%. The median number of VTs was three per patient, mean cycle length of 360 ms (326-400 ms). All patients received endocardial ablation, and 18% required epicardial ablation. From a less strict endpoint (i.e., freedom from arrhythmia or reduction of ventricular arrhythmia burden), 61 (88.4%) patients improved following ablation. There was a relapse in 45 (54.2%) patients. Despite the VT recurrence, ablation is quite effective in treating ventricular arrhythmias in CS patients.

Implantable Cardioverter-Defibrillator (ICD)

ICDs could be used as a prophylactic device to prevent fatal arrhythmias leading to sudden death in CA and CS patients. Arrhythmic sudden death is far more common in AL amyloidosis. In a study involving 53 patients with CA who underwent ICD implantation were included [8]. The rate of appropriate ICD shocks was 32% in the first year and occurred almost exclusively in patients with AL amyloidosis. Appropriate ICD shocks were more frequent in patients with prior sudden cardiac arrest or sustained ventricular arrhythmias. Nevertheless, appropriate ICD therapy did not result in an overall survival benefit. In general, ICDs are not typically given to patients with a life expectancy of less than one year, as is usually the case in cardiac AL amyloidosis, and their application in primary prevention of fatal arrhythmias in CA patients remains uncertain.

ICD implantation is indicated in cardiac sarcoidosis and spontaneous sustained ventricular arrhythmia, particularly those with previous cardiac arrest [2]. According to the 2014 Heart Rhythm Society, class IIb drugs for primary prevention along with implantable cardioverter-defibrillator (ICD) placement are recommended in those patients with mild-to-moderately reduced left ventricular ejection fraction (LVEF 36-49%) and/or reduced right ventricular ejection fraction (RVEF <40%) despite optimal medical therapy and a period of immunosuppression [38]. SCD is not uncommon in CS. The five-year risk of SCD in middle-aged patients is 34% when the AVB is presented with VT or severe LV dysfunction [2]. So, ICD is also recommended in patients with a permanent pacemaker indication due to atrioventricular blocks (AVBs).

## Conclusions

This article outlines the various arrhythmias that occur in cardiac amyloidosis and sarcoidosis. It is important to understand the association of arrhythmias with infiltrative cardiomyopathies, since fatal arrhythmias are the leading cause of death in these diseases.The precise understanding of the mechanism of atrial and ventricular arrhythmias of cardiac amyloidosis remains uncertain. However, several mechanisms, including inflammatory cell damage, cellular degradation, and separation of myocytes by amyloid fibrils, cause CA arrhythmias. Similarly, inflammation and scarring contribute to arrhythmias in cardiac sarcoidosis. Atrial dysfunction resulting in CA due to amyloid accumulation increases thrombus development and raises the likelihood of stroke, which requires a risk assessment. Anticoagulation therapy tends to be protective, but further studies are necessary to determine their function as a prophylactic intervention to prevent stroke in these patients. The CS seems to have become more prevalent, however, this is likely due to improved imaging and/or a more thorough investigation rather than a real increase in prevalence. Corticosteroid reduces the burden of arrhythmias in cardiac sarcoidosis, but then the VT's mechanism is reentry due to scar formation in CS which is less susceptible to corticosteroid recovery.

Further studies are needed to explore the role of corticosteroids and other immunosuppressive drugs in reducing the burden of arrhythmias in cardiac sarcoidosis. Fatal arrhythmias usually lead to sudden death, which is not uncommon in both diseases, and appropriate ICD therapy to prevent these arrhythmias in cardiac amyloidosis has not provided any survival benefit and remains somewhat controversial. In contrast, it is particularly indicated in cardiac sarcoidosis. Sudden death is a disastrous outcome that has to be anticipated, and a better understanding of arrhythmia induced sudden death is required. The early use of ICD should be evaluated in more extensive studies that can help prevent sudden death due to arrhythmia. Ablation treatment only offers symptomatic relief from arrhythmias, so the recurrence risk is very high. Even with recent advancements in medicine, the overall prognosis remains rather dismal.
